# Validation of the Brief Assessment of Stress and Eating (BASE) in cisgender gay men and lesbian women

**DOI:** 10.1186/s40337-025-01482-w

**Published:** 2025-12-05

**Authors:** Jason M. Nagata, Christopher D. Otmar, Ken Murakami, Char Potes, Jason M. Lavender, Emilio J. Compte, Tiffany A. Brown, Kelsie T. Forbush, Annesa Flentje, Juno Obedin-Maliver, Mitchell R. Lunn

**Affiliations:** 1 Department of Pediatrics, University of California, San Francisco, 550 16th Street, 4th Floor, P.O. Box 0503, San Francisco, CA 94143 USA; 2 Military Cardiovascular Outcomes Research Program (MiCOR), Department of Medicine, Uniformed Services University of the Health Sciences, Bethesda, MD USA; 3 The Metis Foundation, San Antonio, TX USA; 4 Eating Behavior Research Center, School of Psychology, Universidad Adolfo Ibáñez, Santiago, Chile; 5 Research Department, Comenzar de Nuevo Treatment Center, Monterrey, México; 6 Department of Psychological Sciences, Auburn University, Auburn, AL USA; 7 Department of Clinical Child Psychology, University of Kansas, Lawrence, KS USA; 8 The PRIDE Study/PRIDEnet, Stanford University School of Medicine, Stanford, CA USA; 9 Stanford Prevention Research Center, Department of Medicine, Stanford University School of Medicine, Stanford, CA USA; 10 Alliance Health Project, Department of Psychiatry and Behavioral Sciences, University of California, San Francisco, San Francisco, CA USA; 11 Department of Obstetrics and Gynecology, Stanford University School of Medicine, Stanford, CA USA; 12 Department of Epidemiology and Population Health, Stanford University School of Medicine, Stanford, CA USA; 13 Division of Nephrology, Department of Medicine, Stanford University School of Medicine, Stanford, CA USA

**Keywords:** Sexual minority, Eating disorder, Brief screeners, Psychometric validation, Health equity

## Abstract

**Background:**

Sexual minority adults are at elevated risk for eating disorders (EDs), yet existing screening tools have rarely been validated in this population. Most ED screening instruments have been validated in predominately cisgender, heterosexual female samples limiting their generalizability to populations with different symptom patterns. Validation studies in cisgender sexual minority (SM) adults are critical to improving detection and addressing disparities in ED identification. The present study evaluated the psychometric performance of the Brief Assessment of Stress and Eating (BASE), a validated 10-item screening tool that assesses DSM-5-aligned eating disorder symptoms and subclinical dysregulated eating behaviors, in a national sample of cisgender gay men and lesbian women.

**Methods:**

Participants were 1,499 cisgender SM adults (61.7% gay men, 38.3% lesbian women) recruited from The PRIDE Study, a U.S.-based longitudinal cohort of sexual and gender minority adults. Respondents completed the BASE, SCOFF questionnaire, and the Eating Disorder Diagnostic Scale-5 (EDDS-5) which we used to derive probable DSM-5 eating disorder (probable ED) status. Receiver operating characteristic (ROC) and precision–recall (PR) curve analyses were conducted to evaluate classification accuracy and identify optimal thresholds.

**Results:**

Both the BASE and SCOFF performed significantly above chance in detecting EDDS-5-derived probable EDs. Among gay men, the BASE (AUC: ROC = 0.785, PRC = 0.702) outperformed the SCOFF (ROC = 0.744, PRC = 0.630). In lesbian women, the two screeners performed similarly (BASE AUC = 0.807; SCOFF AUC = 0.806). Optimal BASE thresholds varied by group with higher sensitivity at lower cutoffs (e.g., ≥ 7).

**Conclusions:**

The BASE provides a reliable, efficient alternative to traditional instruments for screening eating disorders in sexual minority adults, with good performance for identifying EDDS-5–derived probable EDs. Findings support the BASE as a reliable and valid screening tool for use with cisgender SM adults in community, healthcare, and research contexts.

## Abstract (plain language summary)

In the United States, gay men and lesbian women are more likely to experience eating problems than people who identify as heterosexual. Many existing tools for spotting eating disorders were created and tested mainly in heterosexual women, which may limit their usefulness for other groups. One brief tool, the 10-item Brief Assessment of Stress and Eating (BASE), had not previously been tested in gay men and lesbian women. In this study, we examined how well the BASE works in a large national sample of adults who identified as gay men or lesbian women. We found that the BASE was able to reliably detect people who were likely to have eating disorders, and it performed especially well for gay men compared to another widely used tool, the SCOFF questionnaire. These results suggest that the BASE can be a useful and efficient option for identifying eating disorders among sexual minority adults in research, healthcare, and community settings.

## Introduction

Sexual minority (SM) adults remain substantially underrepresented in psychometric studies of eating disorder (ED) screening measures [1], despite consistent evidence that disordered eating behaviors are more prevalent and differently patterned in these populations [2–4]. Compared to cisgender heterosexual adults, cisgender SM adults have two- to four-fold greater odds of meeting DSM-5 criteria for anorexia nervosa, bulimia nervosa, or binge-eating disorder with lifetime prevalence rates of 1.7%, 1.3%, and 2.2%, respectively [5]. Among cisgender gay men, disordered eating symptoms are common with 20% reporting dietary restraint, 11% reporting binge eating, and 10% reporting excessive exercise [6]. Cisgender lesbian women are more likely than gay men to show elevated rates of binge eating and restrictive eating behavior [7]—yet their symptoms are frequently overlooked due to sociocultural misperceptions that EDs primarily affect young, thin, heterosexual females [8]. As a result, existing ED symptom measures may fail to adequately capture the severity and range of eating disorder symptoms and disordered eating behaviors in diverse populations, including SM adults. In this manuscript, we use “eating disorders (EDs)” to refer to DSM-5 diagnoses, “probable ED” to refer to EDDS-5–based classifications, and “disordered eating behaviors” to describe clinically relevant eating-related behaviors that may not meet full diagnostic criteria.

ED screening instruments, in particular, may not account for the ways in which symptoms are shaped by the distinct experiences of cisgender SM adults, such as exposure to stigma-based stress [9, 10] or community-specific appearance expectations [11, 12]. Screeners that emphasize weight phobia and thinness-oriented attitudes may underrepresent ED symptoms in cisgender gay men driven by muscularity-oriented concerns and behaviors [13], or cisgender lesbian women, whose eating pathology frequently involves more binge eating rather than restrictive dieting [14]. As such, efforts to implement screening measures must address structural and interpersonal barriers to diagnosis that disproportionately affect SM adults. Lower trust in providers [21, 22] and higher rates of care avoidance are well-documented across SM populations [23], particularly for concerns related to eating and body image. Thus, self-report questionnaires that require minimal time and do not rely on face-to-face disclosure provide a pragmatic strategy for reaching individuals who may be less likely to disclose symptoms in traditional clinical contexts.

Moreover, lengthy or culturally mismatched measures are associated with lower completion rates [24], and this may be especially true among SM individuals navigating intersecting stigmas related to mental health and sexual identity [25]. Although longer forms are not inherently burdensome [26, 27], their acceptability depends on whether respondents perceive them as relevant and worth the effort [28, 29]. These considerations are particularly relevant to ED screening in community and primary-care contexts, where longer instruments may be less feasible due, in part, to the time constraints of patient-provider interactions [15, 16]. Most primary care visits in the United States last between 13 and 21 min, which leaves little room for preventive mental-health screening or discussion [17]. The limitations of provider training further complicate these challenges. Many clinicians report limited exposure to ED assessment during professional training, lack confidence in identifying atypical or subclinical cases, and express general discomfort initiating conversations about eating and weight concerns, especially when facing uncertainty about what to ask or how to respond [30]. Brief, standardized tools can support clinical judgment by offering accessible entry points for identification and may promote more equitable detection of ED risk across diverse patient populations. These brief, validated measures may also aid in reducing participant burden in ED research studies that involve completion of a variety of additional questionnaires assessing other constructs.

Brief screening tools such as the Sick-Control-One-Fat-Food (SCOFF) [18] and Eating Disorder Examination Questionnaire (EDE-Q7) [19] are available, but like most ED measures, they were developed to reflect the symptom presentations of affluent, cisgender, heterosexual, white females with minimal consideration of how EDs manifest across diverse identities [1]. The Brief Assessment of Stress and Eating (BASE) [31], a more recently developed screening questionnaire, offers a more comprehensive and potentially more inclusive alternative. The 10-item measure assesses multiple symptoms of eating disorders and disordered eating behaviors, including dietary restriction, binge eating, use of muscle-building supplements, excessive exercise, and other compensatory behaviors. Additionally, rather than focusing on weight- or shape-based concerns, the items largely address behavioral indicators of severity, which may make the measure more applicable across individuals with diverse identities and body ideals. The BASE was designed to screen for broadly defined eating pathology [31], including both DSM-aligned symptoms and subclinical behaviors that may not meet full diagnostic criteria, making it suitable for use in community and epidemiological research where early identification is prioritized. Overall, the BASE is brief, simple to administer and score, and preserves symptom breadth while reducing respondent burden.

### Current study

Although the BASE has demonstrated good psychometric performance in college samples [31], its utility in cisgender SM adults remains untested. However, because the BASE was derived from the Eating Pathology Symptoms Inventory (EPSI) [32], which has demonstrated robust internal consistency and measurement invariance across cisgender gay men and lesbian women [33], there is strong rationale to expect adequate performance in this group. Brief, low-burden screening measures (like the BASE) could help address existing disparities by improving detection and enhancing opportunities for prevention or early intervention, particularly in settings where time constraints or limited resources may otherwise preclude comprehensive assessment [34]. These considerations position the BASE as a potentially valuable tool that is feasible and easily administered in a variety of clinical and research settings.

The purpose of the current study was to validate the BASE as a brief screener of eating disorder symptoms and disordered eating behaviors, with a specific focus on its ability to identify EDDS-5–derived probable EDs, in a large community-based sample of cisgender SM adults. Given that the BASE derived items from the Eating Pathology Symptoms Inventory—which has shown evidence for strong psychometric properties in SM adults [33, 35], we hypothesized that the BASE would show good internal consistency and diagnostic accuracy with similar or better performance than the widely used SCOFF questionnaire [18]. This work supports broader efforts to validate psychometrically sound, culturally appropriate instruments for sexual and gender minority populations [33, 36]. Establishing the BASE’s psychometric properties and clinical cut-offs in SM adults is a key step toward improving ED detection and promoting equity.

## Methods

### Participants and procedures

This research was approved by institutional review boards at Stanford University School of Medicine (Protocol #63400), the University of California, San Francisco, and the WIRB-Copernicus Group. Oversight was additionally provided by the Research and Participant Advisory Committees of The Population Research in Identity and Disparities for Equality (PRIDE) Study. The PRIDE Study is a national, longitudinal cohort study centered on understanding the health of sexual and gender minority (SGM) adults residing in the United States and its territories [37, 38]. Participants were eligible for inclusion if they were 18 years of age or older, currently living in the U.S. or its territories, and able to comprehend an English-language survey. Data from the present study came from supplemental data collection focused on eating and body image concerns that was administered between July 2023 and January 2024. Participant outreach involved a range of community-driven and digital methods, including PRIDEnet engagement efforts, announcements in email newsletters and blog posts, visibility at in-person events, targeted social media posts, and informal word-of-mouth referrals. Surveys could be completed from any internet-connected device. To encourage participation, respondents were automatically entered into a drawing for one of fifty $40 gift card.

Survey access was restricted through a secure digital infrastructure. Each individual enrolled in The PRIDE Study was assigned a unique dashboard within the study’s secure portal, from which personalized survey links could be accessed. Once a survey was submitted, it was no longer available to the participant. Each completed response was automatically associated with a unique participant ID generated and tracked by the Qualtrics survey platform.

The current analysis focused on cisgender participants who self-identified as either cisgender gay men or lesbian women. Of the 4,729 individuals who completed the ancillary survey, 1,499 (31.6%) met these inclusion criteria: 925 identified as cisgender gay men (61.7%) and 573 as cisgender lesbian women (38.3%). Eligibility was based on two items: participants selected “gay/lesbian” from a single-choice sexual orientation item and either “cisgender man” or “cisgender woman” from a single-choice gender identity item. The average age of included participants was 50.78 years (SD = 15.2) with responses spanning from 18 to 96 years. The sample was predominantly White (*n* = 1,251, 84.8%), with smaller proportions identifying as Hispanic or Latino (3.6%), Black or African American (2.6%), Asian (2.3%), American Indian or Alaska Native (2.1%), Middle Eastern or North African (0.5%), or Native Hawaiian or Pacific Islander (0.1%). A small proportion of participants (0.5%) reported an ethno-racial identity outside the listed categories or declined to disclose this information. Multiracial identities were reported by 9.3% of the sample, with similar rates across gay men and lesbian women. Most participants had completed a college degree or higher (79.7%), while 20.3% had not. Complete demographic and diagnostic characteristics of the sample are presented in Table 1.

**Table 1:** Demographic and diagnostic characteristics of cisgender sexual minority sample

	Cisgender Gay Men	Cisgender Lesbian Women
	Mean (SD)	Mean (SD)
Age, years	52.8 (15.2)	48.2 (17.1)
BMI, kg/m^2^	28.8 (6.42)	30.9 (8.66)
Ethnoracial identity	n (%)	n (%)
American Indian/Alaska Native	1 (0.86%)	13 (2.90%)
Asian	5 (4.31%)	23 (5.13%)
Black/African American	10 (8.62%)	21 (4.69%)
Hispanic/Latino	7 (6.03%)	27 (6.03%)
Middle Eastern/North African	1 (0.86%)	3 (0.67%)
Native Hawaiian/Pacific Islander	1 (0.86%)	0 (0.00%)
White	102 (87.93%)	416 (92.86%)
Other/Unknown	2 (1.72%)	2 (0.45%)
BMI Category, kg/m^2^	n (%)	n (%)
< 18.50	13 (1.4%)	9 (1.6%)
18.50–24.99	255 (27.7%)	164 (28.8%)
25.00–29.99	316 (34.4%)	132 (23.2%)
≥ 30.00	335 (36.5%)	265 (46.5%)
Education	n (%)	n (%)
No schooling	0 (0.0%)	0 (0.0%)
Nursery to high school, no diploma	1 (0.9%)	0 (0.0%)
High school graduate or equivalent	3 (2.6%)	16 (3.6%)
Trade/Technical/Vocational training	2 (1.7%)	9 (2.0%)
Some college	16 (13.8%)	54 (12.1%)
2-year college degree	4 (3.4%)	28 (6.2%)
4-year college degree	36 (31.0%)	152 (33.9%)
Master’s degree	32 (27.6%)	121 (27.0%)
Doctoral degree	11 (9.5%)	40 (8.9%)
Professional degree	11 (9.5%)	28 (6.2%)
Income	n (%)	n (%)
$0–$30,000	26 (23.2%)	156 (34.8%)
$30,001–$60,000	31 (27.7%)	137 (30.6%)
$60,001–$100,000	25 (22.3%)	97 (21.6%)
$100,001–$150,000	14 (12.5%)	37 (8.3%)
$150,001+	18 (16.1%)	24 (5.3%)
Eating Disorder Diagnosis	n (%)	n (%)
Any eating disorder	338 (36.5%)	216 (37.7%)
Anorexia nervosa	3 (0.3%)	5 (0.9%)
Bulimia nervosa	90 (9.7%)	55 (9.6%)
Binge-eating disorder	10 (1.1%)	20 (3.5%)
OSFED: subthreshold anorexia nervosa	24 (2.6%)	13 (2.3%)
OSFED: subthreshold bulimia nervosa	19 (2.1%)	12 (2.1%)
OSFED: subthreshold binge-eating disorder	22 (2.4%)	42 (7.3%)
OSFED: purging disorder	7 (0.8%)	3 (0.5%)
OSFED: subthreshold purging disorder	35 (3.8%)	14 (2.4%)
OSFED: compensatory eating disorder	148 (16%)	80 (14%)
OSFED: subthreshold compensatory eating disorder	248 (26.8%)	161 (28.1%)

*BMI* body mass index,* OSFED* other specified feeding or eating disorder

## Measures

### Brief Assessment of Stress and Eating (BASE)

The BASE [31] is a 17-item screening questionnaire developed using items from the Eating Pathology Symptoms Inventory (EPSI) [32] and the Inventory for Depression and Anxiety Symptoms–II (IDAS-II) [39]. The BASE is designed to assess core features of EDs, PTSD, depression, and generalized anxiety disorder over the past four weeks. For the current study, only the 10-item ED screen was used. Each item was rated on a 5-point scale ranging from 0 (never) to 4 (very often), with higher values indicating more frequent or severe eating disorder symptoms and disordered eating behaviors.

### Eating Disorder Diagnostic Scale-5

The EDDS-5 [40] is a self-report instrument developed to assess probable DSM-5 ED diagnoses. The original version of the EDDS demonstrated evidence for strong psychometric properties, including high internal consistency, excellent 1-week test-retest reliability for the symptom composite, and good-to-excellent diagnostic agreement with structured clinical interviews. The present study derived probable ED diagnoses using a structured, rule-based algorithm adapted from Stice et al. (2000); [40] to align with DSM-5 diagnostic constructs and permit classification of both full and subthreshold ED presentations. The algorithm generated a set of binary diagnostic flags for each probable *ED* category: anorexia nervosa (full and subthreshold), bulimia nervosa (full and subthreshold), binge-eating disorder (full and subthreshold), purging disorder (full and subthreshold), and compensatory eating disorder. Consistent with earlier BASE validation research (e.g., [31]), we generated a binary composite outcome reflecting whether participants met criteria for a full or subthreshold eating disorder. This composite variable defined probable ED status and served as the criterion outcome in all prediction models. Importantly, subthreshold presentations may fall under other specified feeding and eating disorders (OSFED), which are recognized as clinically significant eating disorders.

### SCOFF questionnaire

The SCOFF questionnaire [18], a five-item screener with binary (yes/no) responses, was administered as comparison screening instrument given its wide application in primary care settings. Following standard scoring procedures, responses to the five items were summed to yield a total score ranging from 0 to 5. The total score was used as a continuous predictor of probable ED status in logistic regression models and was also evaluated as a binary classifier using cutoffs derived from ROC and PR curves to determine optimal thresholds for classification.

### Body mass index

Body mass index (BMI) was calculated using the standard formula: [weight (lbs) / height (in)^2^] × 703, with height reported in feet and inches and converted to inches for calculation. For descriptive analyses, BMI was categorized according to World Health Organization and Centers for Disease Control and Prevention guidelines: underweight (< 18.5 kg/m^2^), normal weight (18.5–24.99 kg/m^2^), overweight (25–29.99 kg/m^2^), and obesity (≥ 30 kg/m^2^) [41, 42]. Separately, a diagnostic low-weight indicator was derived using a three-level classification: <18.5 kg/m^2^ = low weight; 18.5–18.99 kg/m^2^ = subthreshold low weight; and ≥ 19 kg/m^2^ = above low weight threshold. This variable was used within the EDDS-5 scoring algorithm to inform diagnosis.

## Statistical analysis

All analyses were conducted in R (version 4.5.1) [43]. Internal consistency of the BASE was assessed using ordinal alpha, derived from polychoric correlations to account for the ordered categorical format, in the full sample and within SM subgroups. A binary criterion variable for probable ED was created based on available diagnostic data assessed using the EDDS-5 and served as the outcome for all prediction models. For the BASE and SCOFF, total sum scores were utilized, with higher scores indicating greater severity.

Predictive validity was evaluated using binary logistic regression models with BASE total scores as the predictor of probable ED status. Models were fit in the full sample and separately within each subgroup to obtain stratified estimates. Model performance was assessed using receiver operating characteristic (ROC) curve analysis [44]. The area under the ROC curve (AUC ROC) quantified the screener’s ability to distinguish between probable ED cases and non-cases [45, 46]. In addition, precision–recall (PR) curves were generated, and the area under the PR curve (AUC PR) [47] was used as a complementary metric of classifier performance [48], particularly given the presence of class imbalance.

Optimal cut-points were identified using two approaches. First, Youden’s J index was applied to ROC curves based on total scores to determine the score that maximized the joint sensitivity and specificity [49]. Positive and negative predictive values were calculated at this threshold. Second, predicted probabilities from logistic models were used to construct PR curves, from which we extracted the threshold that maximized the F1 score (i.e., the harmonic mean of precision and recall). This approach provided a performance-optimized threshold for practical classification use. To benchmark performance, we conducted parallel analyses using the SCOFF screener. Logistic regression models, ROC and PR curves, and threshold optimization procedures were repeated using SCOFF total scores. DeLong’s test for correlated ROC curves [50] was used to compare the AUCs of the BASE and SCOFF in the full sample and within subgroups.

## Results

In the analytic subsample of cisgender gay men and lesbian women (*N* = 1499; 926 gay men, 573 lesbian women), 36.7% of gay men and 38.0% of lesbian women screened positive for a probable ED on the EDDS-5. Ordinal alpha indicated strong internal consistency in the full sample (α = 0.818), among gay men (α = 0.829), and among lesbian women (α = 0.800); all exceeded the conventional 0.80 threshold for acceptable reliability (see Tables 2 and 3 for BASE descriptive statistics). BASE scores significantly predicted probable ED status across all models. In the full sample, each one-point increase in BASE was associated with 32% higher odds of screening positive for any probable ED (OR = 1.32, 95% CI [1.28, 1.37], *p* < .001). Among lesbian women, the odds were 36% higher (OR = 1.36, 95% CI [1.29, 1.45], *p* < .001); among gay men, the odds were 30% higher (OR = 1.30, 95% CI [1.25, 1.36], *p* < .001).

**Table 2:** Means, Medians, and standard deviations of measures by sexual orientation

	Cisgender gay men		Cisgender lesbian women		Full sample	
Measure	Mean (SD)	Median	Mean (SD)	Median	Mean (SD)	Median
SCOFF	0.83 (1.12)	0	0.95 (1.13)	1	0.87 (1.12)	0
BASE-10-item	8.27 (4.98)	8	7.83 (4.50)	7	8.10 (4.80)	7

**Table 3:** Descriptive statistics for BASE items by group

BASE Item	Cisgender gay men		Cisgender lesbian women		Full sample	
	M (SD)	Med.	M (SD)	Med.	M (SD)	Med.
Does not eat very much	0.61 (0.98)	0	0.70 (0.99)	0	0.65 (0.98)	0
Exercise nearly every day	1.74 (1.46)	2	1.42 (1.42)	1	1.62 (1.45)	2
Muscle building supplements	0.39 (0.92)	0	0.15 (0.58)	0	0.30 (0.81)	0
Body dissatisfaction	2.54 (1.19)	3	2.55 (1.19)	2	2.55 (1.19)	3
Binge eating behavior	1.14 (1.04)	1	1.23 (1.05)	1	1.18 (1.05)	1
Vomit to lose weight	0.07 (0.42)	0	0.08 (0.42)	0	0.08 (0.42)	0
Strenuous exercise (5 + days per week)	0.82 (1.25)	0	0.62 (1.08)	0	0.74 (1.19)	0
Eating until feeling sick	0.51 (0.81)	0	0.65 (0.91)	0	0.56 (0.85)	0
Laxatives or diuretics to lose weight	0.15 (0.60)	0	0.14 (0.54)	0	0.15 (0.58)	0
Substances to reduce hunger	0.28 (0.85)	0	0.29 (0.80)	0	0.28 (0.83)	0

Item summaries have been abbreviated for brevity and to comply with copyright guidance. Full BASE item wording is available at https://care.ku.edu/base

Classification thresholds were derived using Youden’s J statistic. In the full sample, the optimal threshold was 8.5 (sensitivity = 0.68, specificity = 0.76, PPV = 0.62, NPV = 0.80; see Table 4). Subgroup thresholds were 9.5 for gay men and 8.5 for lesbian women with similar diagnostic trade-offs (see Table 5). PR curve optimization selected lower thresholds: 7 in the full sample and gay men, 8 in lesbian women, resulting in increased sensitivity (range = 0.74–0.85) and NPV (0.85–0.86) but decreased specificity (0.54–0.72) and PPV (0.51–0.62), consistent with a shift toward greater case detection at the expense of precision. In the full sample, the BASE showed good overall classification (AUC = 0.79, 95% CI [0.77, 0.82]; AUCPR = 0.71) with comparable results in gay men (AUC = 0.79, AUCPR = 0.70) and slightly stronger performance in lesbian women (AUC = 0.81, AUCPR = 0.73). Although specificity at the Youden-optimal thresholds met conventional benchmarks, sensitivity fell below the ≥ 0.80 threshold favored in clinical contexts, suggesting that lower thresholds may be preferable when minimizing false negatives is a priority [44, 49].

**Table 4:** Predictive accuracy statistics in full sample

	Cutoff-PRC	AUC-PRC	Sensitivity	Specificity	PPV	NPV
SCOFF-optimal	1.5	0.666	0.774	0.683	0.591	0.837
BASE-10-optimal	7	0.711	0.832	0.560	0.528	0.850
BASE-10-9-cutoff	9	0.711	0.681	0.755	0.621	0.800
	Cutoff-ROC	AUC-ROC	Sensitivity	Specificity	PPV	NPV
SCOFF-optimal	1.5	0.768	0.774	0.683	0.591	0.837
BASE-10-optimal	8.5	0.793	0.681	0.755	0.621	0.800
BASE-10-9-cutoff	9	0.793	0.681	0.755	0.621	0.800

We used a BASE-10 cutoff of 9 across both ROC and PRC analyses to maintain consistency with the c threshold established by Forbush et al. [31], where scores ≥ 9 were used to classify probable ED cases in full-sample ROC analyses. Although a lower PRC-optimal threshold (e.g., 7) yielded higher sensitivity in that study, we applied the 9-point cutoff uniformly to enable direct comparisons across screeners and performance metrics.

* AUC* area under the curve, * ROC* receiver operating characteristics,* PRC * precision-recall curve,* PPV* positive predictive value,* NPV * negative predictive value,* ED* eating disorder

**Table 5:** Predictive accuracy statistics by sexual orientation

Cisgender lesbian women						
	Cutoff-PRC	AUC-PRC	Sensitivity	Specificity	PPV	NPV
SCOFF-optimal	1.5	0.722	0.843	0.660	0.603	0.873
BASE-10-optimal	8	0.727	0.736	0.720	0.616	0.817
BASE-10-9-cutoff	9	0.727	0.681	0.796	0.671	0.803
	Cutoff-ROC	AUC-ROC	Sensitivity	Specificity	PPV	NPV
SCOFF-optimal	1.5	0.806	0.843	0.660	0.603	0.873
BASE-10-optimal	8.5	0.807	0.681	0.796	0.671	0.803
BASE-10-9-cutoff	9	0.807	0.681	0.796	0.671	0.803
Cisgender gay men						
	Cutoff-PRC	AUC-PRC	Sensitivity	Specificity	PPV	NPV
SCOFF-optimal	3	0.630	0.731	0.697	0.583	0.817
BASE-10-optimal	7	0.702	0.846	0.538	0.514	0.858
BASE-10-9-cutoff	9	0.702	0.680	0.729	0.593	0.798
	Cutoff-ROC	AUC-ROC	Sensitivity	Specificity	PPV	NPV
SCOFF-optimal	1.5	0.744	0.731	0.697	0.583	0.817
BASE-10-optimal	9.5	0.785	0.601	0.818	0.657	0.780
BASE-10-9-cutoff	9	0.785	0.680	0.729	0.593	0.798

We used a BASE-10 cutoff of 9 across both ROC and PRC analyses to maintain consistency with the c threshold established by Forbush et al. [31], where scores ≥ 9 were used to classify probable ED cases in full-sample ROC analyses.

* AUC* area under the curve, * ROC* receiver operating characteristics,* PRC * precision-recall curve,* PPV* positive predictive value,* NPV * negative predictive value,* ED* eating disorder

**Fig. 1:**
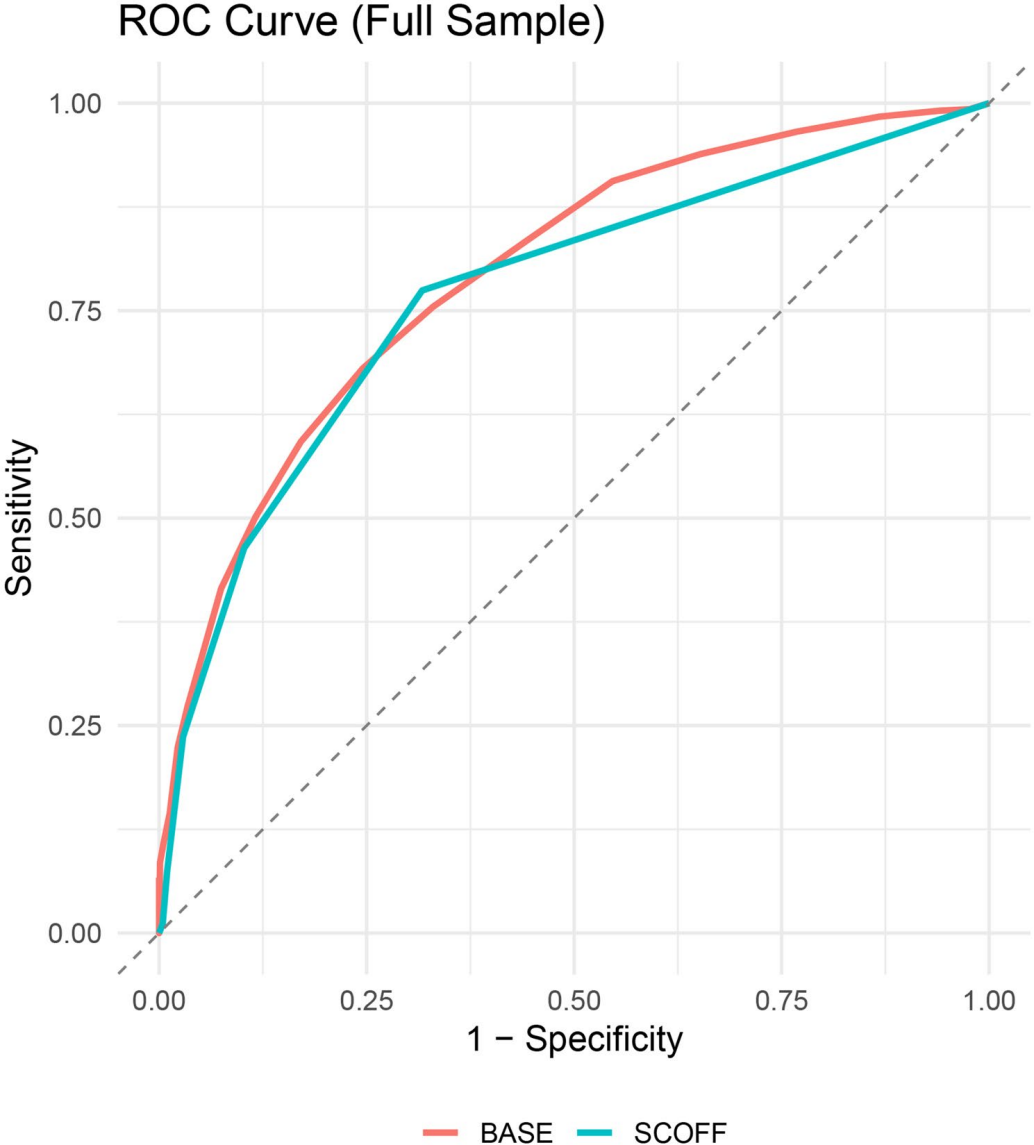
Receiver operating characteristic curves for each screener in the full sample of cisgender sexual minority (SM) adults

**Fig. 2:**
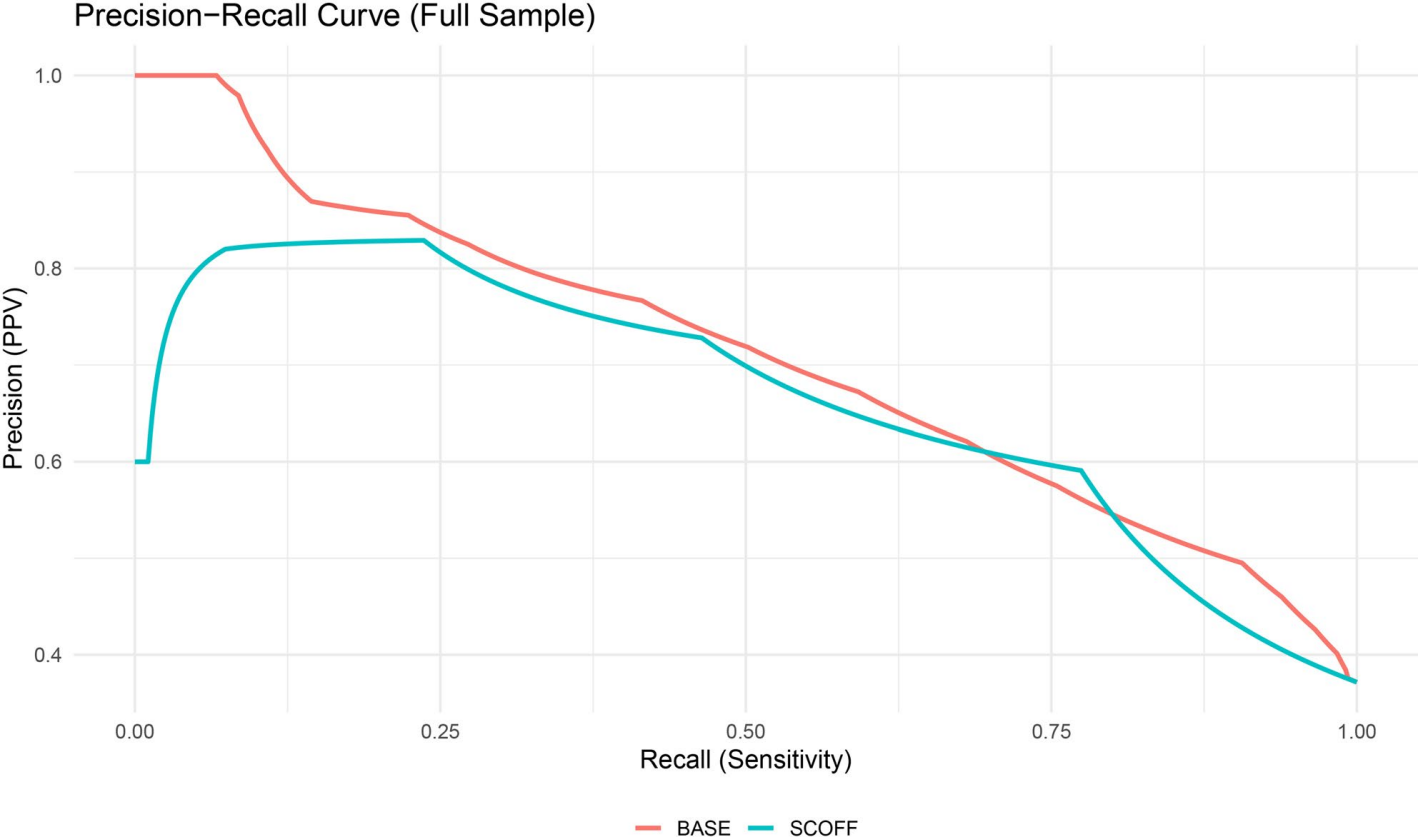
Precision–recall curves for each screener in the full sample of cisgender sexual minority (SM) adults

**Fig. 3:**
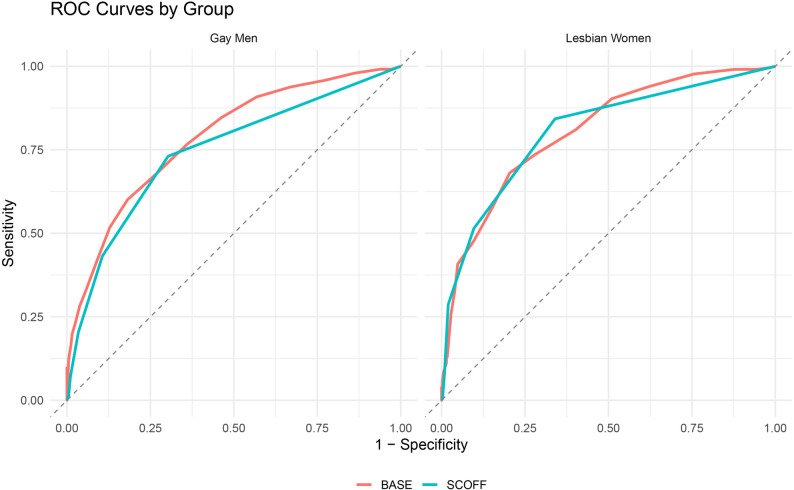
Receiver operating characteristic curves for each screener stratified by cisgender SM subgroup (Lesbian Women, Gay Men)

**Fig. 4:**
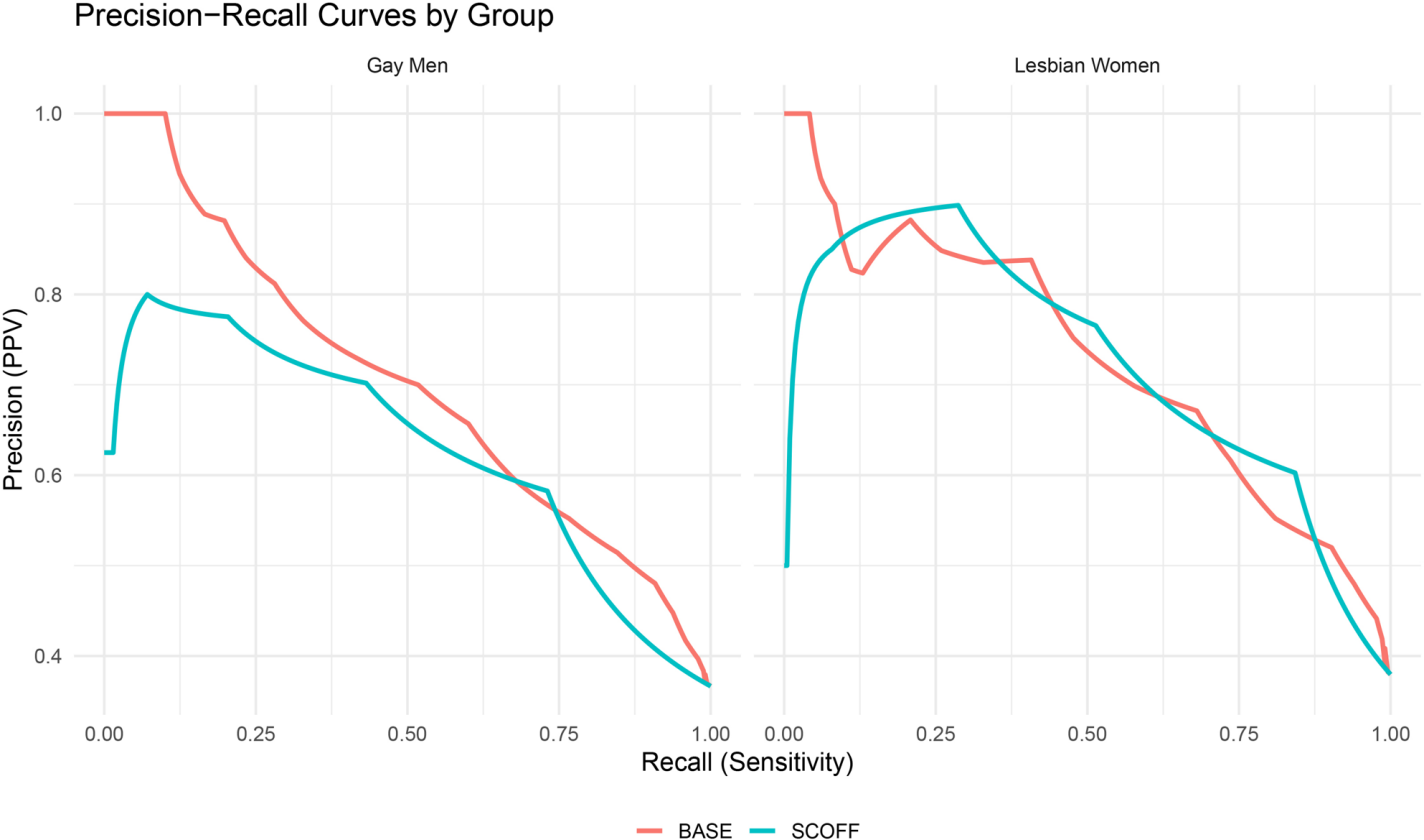
Precision–recall curves for each screener stratified by cisgender SM subgroup (Lesbian Women, Gay Men)

The SCOFF yielded slightly lower accuracy in the full sample (AUC = 0.77, 95% CI [0.74, 0.79]), although the difference from BASE was not statistically significant (Z = 1.83, *p* = .067). Among lesbian women, both screeners performed equivalently (AUC = 0.81; Z = 0.05, *p* = .964). In gay men, however, the BASE outperformed the SCOFF (AUC = 0.79 vs. 0.74; Z = 2.27, *p* = .023). F1-score optimization, which balances precision and recall, yielded peak F1 = 0.65 for BASE (recall = 0.76, precision = 0.58) and F1 = 0.67 for SCOFF (recall = 0.77, precision = 0.59) in the full sample. Among lesbian women, F1 scores were nearly identical (0.70 for SCOFF, 0.68 for BASE); in gay men, the SCOFF again held a marginal advantage (0.65 vs. 0.64), driven by higher recall despite lower precision and specificity. All ROC and PR curves are presented in Figs. 1, 2, 3 and 4, including full-sample plots (Figs. 1 and 2) and subgroup-stratified plots for cisgender sexual minority adults (Figs. 3 and 4).

## Discussion

The present study extends ED screening research by investigating the BASE in a demographically distinct, nationally recruited sample of cisgender SM adults. We sought to determine if the BASE would (1) demonstrate adequate internal consistency reliability; (2) match or exceed the classification accuracy of the SCOFF for probable ED overall; and (3) show utility across SM subgroups. As hypothesized, the BASE exhibited good internal consistency in gay men and lesbian women and separated probable ED cases from non-cases with good overall classification accuracy. In gay men, the BASE outperformed the SCOFF (AUC = 0.79 vs.74); whereas in lesbian women, both screeners performed equivalently. Although the BASE’s classification accuracy was statistically comparable to the SCOFF in the total SM sample and among the cisgender lesbian women subgroup, the BASE achieved superior classification accuracy in the cisgender gay men subgroup. These findings directly address the evidentiary gaps identified by the U.S. Preventive Services Task Force by offering psychometric support for brief, self-administered screening tools in a large cohort of SM adults [20]. In contexts where provider or respondent time is limited and disclosure might be shaped by stigma or uncertainty, the BASE offers a low-burden pathway for identifying individuals with a probable ED in populations historically underserved by traditional screening practices.

The BASE’s sensitivity and specificity in this study were found to fall within, and in some subgroups exceed, the performance range reported for brief ED screeners validated in predominantly cisgender female samples. For instance, the EDE-Q7 has demonstrated AUC values as high as 0.94 in at-risk university samples and sensitivity/specificity above 0.80 in general population studies. In our sample, the BASE achieved comparable discrimination (AUC = 0.79–0.81) for probable ED status and matched the SCOFF in cisgender lesbian women, which mirrored performance estimates from female-majority samples [51, 52]. In cisgender gay men, however, the BASE’s advantage over the SCOFF aligns with literature documenting limited assessment of muscularity-oriented restriction, fasting, and compensatory exercise by traditionally weight loss- and thinness-focused instruments. The inclusion of items that assess compulsive exercise, use of muscle-building supplements, and substance-based appetite control captures symptom patterns that are often underrepresented in traditional ED screeners. These features enhance the cultural responsiveness of the BASE by reflecting behaviors more salient within certain SM subgroups [2], particularly those emphasizing muscularity or performance-oriented body ideals [9]. Items in the BASE that reference compulsive exercise (feeling the need to exercise nearly every day), use of muscle-building supplements, and substance-facilitated appetite control (using medications or substances to reduce hunger or induce weight loss) may explain this differential fit [6, 53].

The BASE achieved AUCPR values around 0.71 which is markedly higher than the 0.62 threshold at which decision-curve analyses begin to yield positive net benefit for probable ED case detection in rare-event contexts. Although sensitivity at the Youden-derived cut-point of 8.5 settled below the ≥ 0.80 threshold favored by most guidelines [54], lowering the threshold to seven boosted recalls to 0.76 while maintaining acceptable negative predictive value. In primary care settings, where minimizing false negatives is critical, a lower threshold (e.g., ≥ 7) may be preferable to maximize detection of individuals with a probable ED. Conversely, epidemiological or large-scale research may prioritize specificity and adopt a higher threshold (e.g., ≥ 9) to reduce false positives. For screening purposes, erring on the side of false positives may be justified when weighed against the costs of prolonged duration of untreated illness, which averages two to three years and predicts poorer clinical outcomes. We therefore recommend referring individuals scoring ≥ 7 for further evaluation for an ED when resources allow, while adopting the more conservative ≥ 9 threshold in settings where confirmatory assessment capacity is limited or infeasible. This recommendation balances flexibility with caution and aligns with the recommended cut-point of ≥ 8 from Forbush et al. [31].

As recommended in efforts to enhance LGBTQIA + cultural sensitivity in health care settings [55], adapting intake and communication processes (including the integration of brief, inclusive tools into electronic health record platforms) can reduce interpersonal risk and support disclosure among patients who may otherwise avoid in-person conversations [56–58]. Given its brevity and broad symptom coverage, the BASE may be well-positioned for use in telehealth or patient portal contexts and in community-based outreach platforms that aim to engage populations historically underserved by traditional clinical care [55]. For researchers, The BASE demonstrates promising psychometric performance in this cross-sectional sample and may offer a feasible tool for initial ED screening for probable EDs in large SM cohort studies.

Several constraints temper the scope of our conclusions. The sample, on average, was older (M = 50.78 years), highly educated, and predominantly White. This demographic profile may limit the extent to which the findings can be generalized to younger and more racially or socioeconomically diverse sexual minority adults whose experiences of risk, stress, and care access may differ in meaningful ways [59, 60]. Younger SM adults, in particular, engage within social environments characterized by heightened appearance-related comparison and digital visibility [61, 62], often coupled with more precarious access to affirming care. It remains an open question whether the BASE performs comparably across age groups. Most prior research on disordered eating among sexual minority populations has centered on adolescents and young adults, whereas the current sample was middle-aged on average. Developmental differences in body image concerns, social comparison processes, and patterns of health care utilization suggest that both item functioning and optimal cut-points could shift across the lifespan. Future studies would benefit from explicitly testing these possibilities, particularly in more racially and economically diverse samples, where the intersecting effects of minority stress, resource constraints, and care barriers are most apparent [63].

Self-selection into The PRIDE Study and this ancillary body image and eating survey may have inflated prevalence estimates; individuals with active concerns could be more inclined to participate despite broad recruitment messaging. Third, probable diagnoses were derived from the self-report EDDS-5 questionnaire rather than structured/semi-structured interviews. Next, the BASE’s performance was evaluated only in cisgender gay men and lesbian women; bisexual, pansexual, and asexual adults often face distinct stigma profiles and body-image pressures that could influence symptom expression [64, 65]. Finally, while PR analyses help mitigate class-imbalance bias, external validation in clinical settings with lower ED prevalence is necessary to establish real-world positive predictive value.

The proportion of participants who screened positive for a probable eating disorder (36.7% of gay men, 38.0% of lesbian women) appears high relative to general population estimates [66–68]. However, this pattern is broadly consistent with prior epidemiologic evidence showing elevated and persistent risk for disordered eating among sexual minority adults, even when sociodemographic factors are taken into account [2–5]. These findings may reflect both genuine differences in underlying vulnerability and the BASE’s capacity to identify subthreshold or behaviorally oriented symptoms not captured by more traditional, weight-focused instruments. From this perspective, the higher prevalence observed may indicate more complete detection rather than inflation, aligning with theoretical accounts that link minority stress processes and chronic stigma exposure to maladaptive regulatory behaviors [69], including disordered eating [10].

Despite these limitations, the current findings represent an incremental but meaningful step forward. The use of The PRIDE Study’s national, community-engaged infrastructure enabled recruitment of a large sample of sexual minority adults without relying on clinic-based pathways, broadening the evidentiary base for ED screening research. Analytically, combining ROC and PR metrics offered a more complete view of accuracy under different prevalence conditions and situated the BASE’s performance relative to a widely used comparator, the SCOFF. Moving forward, the next phase should evaluate the BASE in clinical settings to test its sensitivity across symptom severity, assess test–retest reliability, and determine whether scores predict treatment engagement and recovery outcomes over time. Embedding the tool within primary care workflows would allow examination of feasibility and cost-effectiveness, and randomized implementation trials could clarify whether BASE-guided referrals help reduce the duration of untreated eating disorders.

## Conclusion

The BASE offers a concise, reliable, and diagnostically informative option for identifying probable EDs in cisgender gay men and lesbian women, while also capturing clinically relevant disordered eating behaviors that may not meet full diagnostic criteria. Its superior performance over the SCOFF in cisgender gay men and comparable accuracy in cisgender lesbian women suggest that this brief tool can be an inclusive and useful application in a variety of clinical and research settings when the primary goal is to screen for EDDS-5-derived probable EDs and identify individuals who may benefit from further clinical evaluation.

## Data Availability

Data from The PRIDE Study may be accessed through an Ancillary Study application (details at pridestudy.org/collaborate).

## References

[1] Alexander T, Burnette CB, Cory H, McHale S, Simone M. The need for more inclusive measurement to advance equity in eating disorders prevention. Eat Disord. 2024;32:798–816. 10.1080/10640266.2024.2328460. 38488765 10.1080/10640266.2024.2328460 PMC11401964

[2] Calzo JP, Blashill AJ, Brown TA, Argenal RL. Eating disorders and disordered weight and shape control behaviors in sexual minority populations. Curr Psychiatry Rep. 2017;19:49. 10.1007/s11920-017-0801-y. 28660475 10.1007/s11920-017-0801-y PMC5555626

[3] Feldman MB, Meyer IH. Eating disorders in diverse lesbian, gay, and bisexual populations. Int J Eat Disord. 2007;40:218–26. 10.1002/eat.20360. [doi]. 17262818 10.1002/eat.20360 PMC2080655

[4] Kamody RC, Grilo CM, Udo T. Disparities in DSM-5 defined eating disorders by sexual orientation among U.S. adults. Int J Eat Disord. 2020;53:278–87. 10.1002/eat.23193. 31670848 10.1002/eat.23193

[5] Nagata JM, Ganson KT, Austin SB. Emerging trends in eating disorders among sexual and gender minorities. Curr Opin Psychiatry. 2020;33:562–7. 10.1097/YCO.0000000000000645. 32858597 10.1097/YCO.0000000000000645 PMC8060208

[6] Nagata JM, Capriotti MR, Murray SB, Compte EJ, Griffiths S, Bibbins-Domingo K, et al. Community norms for the eating disorder examination questionnaire among cisgender gay men. Eur Eat Disorders Rev. 2020;28:92–101. 10.1002/erv.2708. 10.1002/erv.2708 PMC7275693 31793119

[7] Nagata JM, Otmar CD, Lee CM, Compte EJ, Lavender JM, Brown TA, et al. Community norms of the Eating Pathology Symptoms Inventory (EPSI) in cisgender sexual minority adults. Eat Weight Disord. 2025;30:34. 10.1007/s40519-025-01742-3. 40183833 10.1007/s40519-025-01742-3 PMC11971188

[8] Jones R, Malson H. A critical exploration of lesbian perspectives on eating disorders. Psychol Sexuality Routledge. 2013;4:62–74. 10.1080/19419899.2011.603349.

[9] Convertino AD, Helm JL, Pennesi J-L, Gonzales IVM, Blashill AJ. Integrating minority stress theory and the tripartite influence model: a model of eating disordered behavior in sexual minority young adults. Appetite. Netherlands: Elsevier Science; 2021. p. 163. 10.1016/j.appet.2021.105204. 10.1016/j.appet.2021.105204 33741450

[10] Santoniccolo F, Rollè L. The role of minority stress in disordered eating: a systematic review of the literature. Eat Weight Disord. 2024;29:41. 10.1007/s40519-024-01671-7. 38850334 10.1007/s40519-024-01671-7 PMC11162380

[11] Denning DM, Billman Miller MG, Kakar V, Yogaratnam N, Johnson N, Brown TA. Examining the prospective effects of multiple sources of minority stress on disordered eating in sexual minority men of color. Stigma and Health. US: Educational Publishing Foundation; 2025. 10.1037/sah0000646.

[12] Pachankis JE, Clark KA, Burton CL, Hughto JMW, Bränström R, Keene DE. Sex, status, competition, and exclusion: intraminority stress from within the gay community and gay and bisexual men’s mental health. J Pers Soc Psychol. 2020;119:713–40. 10.1037/pspp0000282. 31928026 10.1037/pspp0000282 PMC7354883

[13] Yelland C, Tiggemann M. Muscularity and the gay ideal: body dissatisfaction and disordered eating in homosexual men. Eat Behav. 2003;4:107–16. 10.1016/S1471-0153(03)00014-X. 15000974 10.1016/S1471-0153(03)00014-X

[14] Dotan A, Bachner-Melman R, Dahlenburg SC. Sexual orientation and disordered eating in women: a meta-analysis. Eat Weight Disord. 2021;26:13–25. 10.1007/s40519-019-00824-3. 31797331 10.1007/s40519-019-00824-3

[15] Dugdale DC, Epstein R, Pantilat SZ. Time and the patient-physician relationship. J Gen Intern Med. 1999;14(Suppl 1). 10.1046/j.1525-1497.1999.00263.x. :S34-40. 10.1046/j.1525-1497.1999.00263.x PMC1496869 9933493

[16] Ogden J, Bavalia K, Bull M, Frankum S, Goldie C, Gosslau M, et al. I want more time with my doctor: a quantitative study of time and the consultation. Fam Pract. 2004;21:479–83. 10.1093/fampra/cmh502. 15367468 10.1093/fampra/cmh502

[17] Neprash HT, Mulcahy JF, Cross DA, Gaugler JE, Golberstein E, Ganguli I. Association of primary care visit length with potentially inappropriate prescribing. JAMA Health Forum. 2023;4:e230052. 10.1001/jamahealthforum.2023.0052. 36897582 10.1001/jamahealthforum.2023.0052 PMC10249052

[18] Morgan JF, Reid F, Lacey JH. The SCOFF questionnaire: assessment of a new screening tool for eating disorders. BMJ. 1999;319:1467–8. 10.1136/bmj.319.7223.1467. 10582927 10.1136/bmj.319.7223.1467 PMC28290

[19] Rand-Giovannetti D, Cicero DC, Mond JM, Latner JD. Psychometric properties of the Eating Disorder Examination-Questionnaire (EDE-Q): a confirmatory factor analysis and assessment of measurement invariance by sex. Assessment. 2020;27:164–77. 10.1177/1073191117738046. 29094603 10.1177/1073191117738046

[20] US Preventive Services Task Force, Davidson KW, Barry MJ, Mangione CM, Cabana M, Chelmow D, et al. Screening for eating disorders in adolescents and adults: US preventive services task force recommendation statement. JAMA. 2022;327:1061–7. 10.1001/jama.2022.1806. 35289876 10.1001/jama.2022.1806

[21] Cox AB, Jaiswal J, LoSchiavo C, Witte T, Wind S, Griffin M, et al. Medical mistrust among a racially and ethnically diverse sample of sexual minority men. LGBT Health. 2023;10:471–9. 10.1089/lgbt.2022.0252. 37418567 10.1089/lgbt.2022.0252 PMC10623470

[22] Sajwani A, Whitton SW, Swann G, Newcomb ME. Factors associated with medical mistrust among sexual and gender minority young adults. Sex Res Soc Policy [Internet]. 2025. 10.1007/s13178-025-01139-y. [cited 2025 Jul 11]. 10.1007/s13178-025-01139-y PMC12971892 41815234

[23] Liu M, Patel VR, Sandhu S, Reisner S, Keuroghlian AS. Health care discrimination and care avoidance due to patient-clinician identity discordance among sexual and gender minority adults. Ann Fam Med. 2024;22:329–32. 10.1370/afm.3130. 39038968 10.1370/afm.3130 PMC11268683

[24] Bryant E, Spielman K, Le A, Marks P, National Eating Disorder Research Consortium, Touyz S, et al. Screening, assessment and diagnosis in the eating disorders: findings from a rapid review. J Eat Disord. 2022;10:78. 10.1186/s40337-022-00597-8. 35672777 10.1186/s40337-022-00597-8 PMC9175461

[25] Jenkins WD, Walters S, Phillips G, Green K, Fenner E, Bolinski R, et al. Stigma, mental health, and health care use among rural sexual and gender minority individuals. Health Educ Behav. 2024;51:477–89. 10.1177/10901981221120393. 36036544 10.1177/10901981221120393 PMC10064479

[26] Ettridge K, Caruso J, Roder D, Prichard I, Scharling-Gamba K, Wright K, et al. A randomised online experimental study to compare responses to brief and extended surveys of health-related quality of life and psychosocial outcomes among women with breast cancer. Qual Life Res. 2021;30:407–23. 10.1007/s11136-020-02651-x. 32990882 10.1007/s11136-020-02651-x

[27] Rolstad S, Adler J, Rydén A. Response burden and questionnaire length: is shorter better? A review and meta-analysis. Value Health. 2011;14:1101–8. 10.1016/j.jval.2011.06.003. 22152180 10.1016/j.jval.2011.06.003

[28] Aiyegbusi OL, Cruz Rivera S, Roydhouse J, Kamudoni P, Alder Y, Anderson N, et al. Recommendations to address respondent burden associated with patient-reported outcome assessment. Nat Med. 2024;30:650–9. 10.1038/s41591-024-02827-9. 38424214 10.1038/s41591-024-02827-9

[29] Cruz Rivera S, Aiyegbusi OL, Ives J, Draper H, Mercieca-Bebber R, Ells C, et al. Ethical considerations for the inclusion of patient-reported outcomes in clinical research: the PRO ethics guidelines. JAMA. 2022;327:1910–9. 10.1001/jama.2022.6421. 35579638 10.1001/jama.2022.6421

[30] Linville D, Benton A, O’Neil M, Sturm K. Medical providers’ screening, training and intervention practices for eating disorders. Eat Disord. 2010;18:110–31. 10.1080/10640260903585532. 20390615 10.1080/10640260903585532

[31] Forbush KT, Richson BN, Swanson TJ, Thomeczek ML, Negi S, Johnson SN, et al. Screening for eating disorders across genders in college students: initial validation of the brief assessment of stress and eating. Int J Eat Disord. 2022;55:1553–64. 10.1002/eat.23815. 36135594 10.1002/eat.23815 PMC10044497

[32] Forbush KT, Wildes JE, Pollack LO, Dunbar D, Luo J, Patterson K, et al. Development and validation of the Eating Pathology Symptoms Inventory(EPSI). Psychol Assess. 2013;25:859–78. 10.1037/a0032639. 23815116 10.1037/a0032639

[33] Nagata JM, Otmar CD, Kim AE, Compte EJ, Lavender JM, Brown TA, et al. Factor structure, internal consistency, and measurement invariance of the eating pathology symptoms inventory (EPSI) in a National U.S. Sample of cisgender gay men and lesbian women. J Eat Disord. 2025;13:83. 10.1186/s40337-025-01277-z. 40369683 10.1186/s40337-025-01277-z PMC12076874

[34] Attia E, Guarda AS. Prevention and early identification of eating disorders. JAMA. 2022;327:1029–31. 10.1001/jama.2022.2458. 35289893 10.1001/jama.2022.2458

[35] Perko VL, Forbush KT, Christensen KA, Richson BN, Chapa DAN, Bohrer BK, et al. Validation of the factor structure of the eating pathology symptoms inventory in an international sample of sexual minority men. Eat Behav. 2021;42:101511. 10.1016/j.eatbeh.2021.101511. 34004456 10.1016/j.eatbeh.2021.101511 PMC10042082

[36] Nagata JM, Otmar CD, Lopez A, Compte EJ, Lavender JM, Brown TA, et al. Factor structure, internal consistency, and measurement invariance of the eating pathology symptoms inventory (EPSI) in transgender and gender-expansive adults. Int J Eat Disorders [Internet]. 2025. 10.1002/eat.24433. 10.1002/eat.24433 PMC12227293 40183494

[37] Lunn MR, Lubensky M, Hunt C, Flentje A, Capriotti MR, Sooksaman C, et al. A digital health research platform for community engagement, recruitment, and retention of sexual and gender minority adults in a national longitudinal cohort study–The PRIDE study. J Am Med Inf Association: JAMIA. 2019. 10.1093/jamia/ocz082. 10.1093/jamia/ocz082 PMC6696499 31162545

[38] Obedin-Maliver J, Hunt C, Flentje A, Armea-Warren C, Bahati M, Lubensky ME et al. Engaging sexual and gender minority (SGM) communities for health research: Building and sustaining PRIDEnet. J Community Engagem Scholarsh [Internet]. 2024 [cited 2024 Jul 25];16. 10.54656/jces.v16i2.484 10.54656/jces.v16i2.484 PMC11326444 39149568

[39] Watson D, O’Hara MW, Naragon-Gainey K, Koffel E, Chmielewski M, Kotov R, et al. Development and validation of new anxiety and bipolar symptom scales for an expanded version of the IDAS (the IDAS-II). Assessment. 2012;19:399–420. 10.1177/1073191112449857. 22822173 10.1177/1073191112449857

[40] Stice E, Fisher M, Martinez E. Eating disorder diagnostic scale: additional evidence of reliability and validity. Psychol Assess. 2004;16:60–71. 10.1037/1040-3590.16.1.60. 15023093 10.1037/1040-3590.16.1.60

[41] Centers for Disease Control. Adult BMI categories [Internet]. BMI. 2024 [cited 2025 Jul 10]. https://www.cdc.gov/bmi/adult-calculator/bmi-categories.html. Accessed 10 Jul 2025.

[42] World Health Organization. A healthy lifestyle - WHO recommendations [Internet]. 2010 [cited 2025 Jul 11]. https://www.who.int/europe/news-room/fact-sheets/item/a-healthy-lifestyle---who-recommendations. Accessed 11 Jul 2025.

[43] R Core Team. R: a language and environment for statistical computing (Version 4.5.1) [Internet]. R Foundation for Statistical Computing. 2025. https://www.R-project.org/

[44] Hajian-Tilaki K. Receiver Operating Characteristic (ROC) curve analysis for medical diagnostic test evaluation. Casp J Intern Med. 2013;4:627–35. PMC3755824 24009950

[45] Faraggi D, Reiser B. Estimation of the area under the ROC curve. Stat Med. 2002;21:3093–106. 10.1002/sim.1228. 12369084 10.1002/sim.1228

[46] Hsiao JK, Bartko JJ, Potter WZ. Diagnosing diagnoses. Receiver Operating Characteristic methods and psychiatry. Arch Gen Psychiatry. 1989;46:664–7. 10.1001/archpsyc.1989.01810070090014. 2735814 10.1001/archpsyc.1989.01810070090014

[47] Cook J, Ramadas V. When to consult precision-recall curves. Stata J SAGE Publications. 2020;20:131–48. 10.1177/1536867X20909693.

[48] Davis J, Goadrich M. The relationship between Precision-Recall and ROC curves. Proceedings of the 23rd International Conference on Machine Learning. 2006. pp. 233–40.

[49] Fluss R, Faraggi D, Reiser B. Estimation of the Youden index and its associated cutoff point. Biom J. 2005;47:458–72. 10.1002/bimj.200410135. 16161804 10.1002/bimj.200410135

[50] DeLong ER, DeLong DM, Clarke-Pearson DL. Comparing the areas under two or more correlated receiver operating characteristic curves: a nonparametric approach. Biometrics. 1988;44:837–45. 3203132

[51] Meule A, Hilbert A, de Zwaan M, Brähler E, Koch S, Voderholzer U. Cutoff scores of the Eating Disorder Examination-Questionnaire for the German population. Int J Eat Disord. 2024;57:602–10. 10.1002/eat.24133. 38258314 10.1002/eat.24133

[52] Wade T, Pennesi J-L, Zhou Y. Ascertaining an efficient eligibility cut-off for extended medicare items for eating disorders. Australas Psychiatry. 2021;29:519–22. 10.1177/10398562211028632. 34266291 10.1177/10398562211028632

[53] Nagata JM, McGuire FH, Lavender JM, Brown TA, Murray SB, Compte EJ, et al. Appearance and performance-enhancing drugs and supplements (APEDS): lifetime use and associations with eating disorder and muscle dysmorphia symptoms among cisgender sexual minority people. Eating behaviors. Eat Behav. 2022;44:101595. 10.1016/J.EATBEH.2022.101595. 35066385 10.1016/j.eatbeh.2022.101595 PMC9359347

[54] Hosmer DW, Lemeshow S, Sturdivant R. Applied logistic regression. Wiley; 2013.

[55] Ricca P, Wahlskog C, Bergren MD. Enhancing cultural sensitivity in a community health care setting for LGBTQ patients. J Community Health Nurs. 2018;35:165–78. 10.1080/07370016.2018.1516420. 30285486 10.1080/07370016.2018.1516420

[56] Datta N, Derenne J, Sanders M, Lock JD. Telehealth transition in a comprehensive care unit for eating disorders: challenges and long-term benefits. Int J Eat Disord. 2020;53:1774–9. 10.1002/eat.23348. 32715512 10.1002/eat.23348

[57] Lyles CR, Nelson EC, Frampton S, Dykes PC, Cemballi AG, Sarkar U. Using electronic health record portals to improve patient engagement: research priorities and best practices. Ann Intern Med. 2020;172:S123–9. 10.7326/M19-0876. 32479176 10.7326/M19-0876 PMC7800164

[58] Zimmer D, Staab EM, Ridgway JP, Schmitt J, Franco M, Hunter SJ, et al. Population-level portal-based anxiety and depression screening perspectives in HIV care clinicians: qualitative study using the consolidated framework for implementation research. JMIR Form Res. 2024;8:e48935. 10.2196/48935. 38206651 10.2196/48935 PMC10811578

[59] Hsieh N, Ruther M. Sexual minority health and health risk factors: intersection effects of gender, race, and sexual identity. Am J Prev Med. 2016;50:746–55. 10.1016/j.amepre.2015.11.016. 26803358 10.1016/j.amepre.2015.11.016 PMC4875806

[60] Dahlhamer JM, Galinsky AM, Joestl SS, Ward BW. Barriers to health care among adults identifying as sexual minorities: a US national study. Am J Public Health. 2016;106:1116–22. 10.2105/AJPH.2016.303049. 26985623 10.2105/AJPH.2016.303049 PMC4880242

[61] Ramirez A, Rivera DB, Cerezo A. Social media and social connection in sexual and gender minority young adults. Couns Psychol SAGE Publications Inc. 2025;53:523–51. 10.1177/00110000251359690.

[62] Escobar-Viera C, Shensa A, Hamm M, Melcher EM, Rzewnicki DI, Egan JE, et al. I don’t feel like the odd one: utilizing content analysis to compare the effects of social media use on well-being among sexual minority and nonminority US young adults. Am J Health Promot. 2020;34:285–93. 10.1177/0890117119885517. 31698919 10.1177/0890117119885517 PMC7404611

[63] Burke NL, Schaefer LM, Hazzard VM, Rodgers RF. Where identities converge: the importance of intersectionality in eating disorders research. Int J Eat Disord. 2020;53:1605–9. 10.1002/eat.23371. 32856342 10.1002/eat.23371 PMC7722117

[64] Feinstein BA, Dyar C. Bisexuality, minority stress, and health. Curr Sex Health Rep. 2017;9:42–9. 10.1007/s11930-017-0096-3. 28943815 10.1007/s11930-017-0096-3 PMC5603307

[65] Schneckenburger SA, Tam MWY, Ross LE. Asexual competent practices in healthcare: a narrative review. J Gay Lesbian Mental Health United Kingdom: Taylor Francis. 2024;28:314–34. 10.1080/19359705.2023.2214528.

[66] Ward ZJ, Rodriguez P, Wright DR, Austin SB, Long MW. Estimation of eating disorders prevalence by age and associations with mortality in a simulated nationally representative US cohort. JAMA Netw Open. 2019;2:e1912925. 10.1001/jamanetworkopen.2019.12925. 31596495 10.1001/jamanetworkopen.2019.12925 PMC6802241

[67] Udo T, Grilo CM. Prevalence and correlates of DSM-5-defined eating disorders in a nationally representative sample of U.S. adults. Biological psychiatry. Elsevier USA. 2018;84:345–54. 10.1016/j.biopsych.2018.03.014. 10.1016/j.biopsych.2018.03.014 PMC6097933 29859631

[68] Qian J, Wu Y, Liu F, Zhu Y, Jin H, Zhang H, et al. An update on the prevalence of eating disorders in the general population: a systematic review and meta-analysis. Eat Weight Disord. 2022;27:415–28. 10.1007/s40519-021-01162-z. 33834377 10.1007/s40519-021-01162-z PMC8933366

[69] Frost DM, Meyer IH. Minority stress theory: application, critique, and continued relevance. Curr Opin Psychol. 2023;51:101579. 10.1016/j.copsyc.2023.101579. 37270877 10.1016/j.copsyc.2023.101579 PMC10712335

